# Supplementary material for: Understanding American premium chocolate consumer perception of craft chocolate and desirable product attributes using focus groups and projective mapping
Source: PLoS One. 2020 Nov 4;15(11):e0240177. doi: 10.1371/journal.pone.0240177 (PMC7641343; doi:10.1371/journal.pone.0240177)
Supplement: S1 Appendix — (PDF) [file pone.0240177.s001.pdf]

S1 Appendix Focus Group Moderator Guide

Introduction

Good morning and welcome to our session. Thanks for taking the time to join us to talk about chocolate. My name is Allison and joining me is Helene. We're both with Penn State University. We are researching chocolate consumers and are interested to know what chocolate consumers are looking for in chocolate products.

You were invited because you matched our criteria for chocolate consumption habits.

There are no wrong answers but rather differing points of view. Please feel free to share your point of view even if it differs from what others have said

Our Ground Rules—Before we get started, we have some Ground Rules. They are also posted on the wall.

1. Phones off: Please turn your cell phones off so we don't get interrupted.
2. First Names only: Please use your first name only. If two of you have the same name, we may put your last name initial on your nametag
3. Everyone's Opinion Counts: Everyone's opinion is welcome – there are no right or wrong answers; all experiences and opinions are welcome and help us learn
4. Respect one another: it's okay to disagree; it's not okay to put anyone down
5. You're being audio recorded: The research team is very interested in what you have to say. For research purposes, we are recording this discussion using the audio recorder on the table. In order for all of your opinions to be heard it is important that we speak one at a time. These recordings are not shared with anyone outside the research team and are destroyed at the end of the study. The final notes do not include your names.
6. You're being observed and the observer may be chiming in at different times throughout the study.
7. Speak your Mind: The research team and I need your honest feelings on these products and we are okay with whatever opinions you have. Your truthfulness is what we really need to be successful today.

Introductions
Well, let's begin. You're each wearing nametags. Let's find out some more about each other by going around the table. Tell us your name and describe your most memorable chocolate moment.

Questions
First set of questions—Homework assignment: Do show and tell of homework assignment
1. Did everyone recognize these chocolates?
2. Which chocolates do you buy for yourself?
3. Were there some you'd never seen before? Which ones?
4. Would you like to see them in real-life?
5. What made you decide to put your different chocolates in different locations?
6. What made you decide to label or group your chocolates as you did?

Second set of questions—Products
Show Hershey bar, taste it:
1. What words would you use to describe this product?
2. What elements of this product do you find appealing, unappealing (packaging, flavor, size, shape)?

Show Lindt bar, taste it:
1. What words would you use to describe this product?
2. What elements of this product do you find appealing, unappealing (packaging, flavor, size, shape)?

Show Green & Black, taste it:
1. What words would you use to describe this product?
2. What elements of this product do you find appealing, unappealing (packaging, flavor, size, shape)?
3. Note that this package has a fair trade label and an organic label. Is that appealing or unappealing? Would the presence of these labels make you more likely or unlikely to purchase this chocolate?

Show Rainforest Bar, taste it:
1. What words would you use to describe this product?
2. What elements of this product do you find appealing, unappealing (packaging, flavor, size, shape)?
3. Note that this package has a fair trade label and a GMO-free label. Is that appealing or unappealing? Would the presence of these labels make you more likely or unlikely to purchase this chocolate?

Show Dandelion bar, taste it:
1. What words would you use to describe this product?
2. What elements of this product do you find appealing, unappealing (packaging, flavor, size, shape)?
3. Did anyone notice it said Madagascar on this? Why is it on this bar?

Third set of questions

1. What encourages you to purchase a new food product that you have not seen before (e.g. family/friend recommendation, in-store display, in-store sample)?
2. If you were going to tell a friend to find these chocolate, what would you tell them?
3. If you would have to put one word on these chocolates, what would it be?

Optional questions (if time allows)—purchasing habits

1. Is chocolate a planned purchase (i.e. you know you will purchase) or do you purchase chocolate products on impulse when you see them?

2. Do you buy chocolate products regularly (during weekly trips to the grocery store) or for special occasions (e.g. Valentine's Day, Halloween)?

3. Who, in your household, eats chocolate products (e.g. you, other adults, children)?

4. From which retail outlet do you primarily purchase chocolate products (e.g. supermarket, warehouse club)?

Concluding thoughts:

- Any final comments that you would like to express or questions that you would like to ask?

- **Alyssa and Helene** is there anything additional you'd like to ask or add?

- Thank you for attending the focus group. We ask that you do not share any specifics that we discussed with others as we will be continuing our research and conducting additional studies.
